# Aberrant right subclavian artery: case report and literature review

**DOI:** 10.1590/1677-5449.202300832

**Published:** 2025-06-09

**Authors:** Ana Gabriela Fernandes Peixoto Martins, Marco Rafael Lopes da Cunha

**Affiliations:** 1 Unidade de Saúde Familiar Nós e Vós Saúde, Fafe, Portugal.; 2 Unidade de Saúde Familiar São Lourenço, Braga, Portugal.

Dear editor,

Nasser et al. reported a very interesting case of an 82-year-old asymptomatic female with an aberrant right subclavian artery (ARSA) which was treated clinically.^1^ In fact, according to recent literature, only symptomatic patients should undergo surgical treatment, whereas asymptomatic patients should be treated clinically with hypertension control, antiaggregants, and cholesterol reducers.^2^

As described in the literature, ARSA is a rare condition that is more common in women than in men and, when symptomatic, can cause several symptoms namely dysphagia, coughing, and thoracic pain due to compression of the esophagus and the trachea. Other complications may occur, namely the Kommerell diverticulum and occlusive atherosclerotic disease.^1,3^

In our practice as family medicine doctors we also observed a similar case to the one described by Nasser et al. We observed a 75-year-old asymptomatic female, with hypertension and high cholesterol levels, medicated with Perindopril 10mg and Sinvastatine 40 mg in our routine consultation. Objectively, the patient presented blood pressure of 229/98 mmHg in her left arm, 109/74 mmHg in her right arm, 242/80 mmHg in her left leg, and 232/100mmHg in her right leg and a low amplitude left radial pulse.

The patient was immediately referred to the emergency service at the reference hospital, where a thoracoabdominal CT scan revealed a retro-esophageal ARSA. The patient was hospitalized in order to further study her condition, since extensive atherosclerotic disease with 80-85% stenosis of the initial portion of both internal carotid arteries had been detected. Because the patient was asymptomatic, she was discharged from the hospital with antihypertensives and antiplatelet drugs, as in the case reported by Nasser et al.^1^

As family medicine doctors, we should be alert to the importance of maintaining good semiological practice, measuring blood pressure in both arms, and must be vigilant to any sign or symptom that might be caused by ARSA.

## References

[1] Nasser M, Petrocheli BB, Felippe TKS (2023). Aberrant right subclavian artery: case report and literature review. J Vasc Bras.

[2] Domínguez-Massa C, Berbel-Bonillo A, Pérez-Guillen M, Montero-Argudo JA (2019). Dissected aberrant right subclavian artery with Kommerell diverticulum. Rev Port Cardiol.

[3] Pessuti F, Fontes CAP (2019). Divertículo de Kommerell: rara etiologia de disfagia. Rev Fac Ciênc Méd Sorocaba..

