# Supplementary material for: Aberrant right subclavian artery: case report and literature review
Source: J Vasc Bras. 2025 Jun 9;24:e20230083. doi: 10.1590/1677-5449.202300832 (PMC12186680; doi:10.1590/1677-5449.202300832)
Supplement: Related texts

# Reply: Response Letter

**Authors:** Michel Nasser, Bruna Beatriz Petrocheli, Thais Keltke Santos Felippe, Beatriz Isola, Beatriz Caroline dos Santos Pereira, Ana Luiza Carvalho Sartoreli, João Marques Batista, Gustavo Muçouçah Sampaio Brandão

**Affiliations:** 1 Universidade Federal de São Carlos – UFSCar, Departamento de Medicina, São Carlos, SP, Brasil.

Dear editor,

Publication of case reports is a rapid and effective means of disseminating medical information, making it crucial for opportune interventions. Diagnosis and treatment of an aberrant right subclavian artery must be individualized according to the peculiarities of each patient and of the condition, since, despite being the most common anomaly of the aortic arch, it is not easy to find management guidelines in the medical literature^1^. As scientific output progresses, we hope that in the near future we can increase the body of available evidence on the subject and this will only be possible thanks to contributions such as those made by yourselves. We are therefore grateful for your valuable contributions.

## References

[1] Pessuti F, Fontes CAP (2019). Divertículo de Kommerell: rara etiologia de disfagia. Rev Fac Ciênc Méd Sorocaba..
